# Biogenic Synthesis of Calcium-Based Powders from Marine Mollusk Shells: Comparative Characterization and Antibacterial Potential

**DOI:** 10.3390/ma18143331

**Published:** 2025-07-15

**Authors:** Adriana-Gabriela Schiopu, Mihai Oproescu, Alexandru Berevoianu, Raluca Mărginean, Laura Ionașcu, Viorel Năstasă, Andra Dinache, Paul Mereuță, Kim KeunHwan, Daniela Istrate, Adriana-Elena Bălan, Stefan Mira

**Affiliations:** 1 Faculty of Mechanics and Technology, Pitesti University Centre, National University of Science and Technology POLITEHNICA Bucharest, 110040 Pitesti, Romania; gabriela.schiopu@upb.ro (A.-G.S.); stefanmira05@gmail.com (S.M.); 2 Faculty of Electronics, Communication and Computers, Pitesti University Centre, National University of Science and Technology POLITEHNICA Bucharest, 110040 Pitesti, Romania; 3 Horia Hulubei National Institute for R&D in Physics and Nuclear Engineering (IFIN-HH), 077125 Magurele, Romania; raluca.marginean@nipne.ro (R.M.); laura_ionascu@nipne.ro (L.I.); viorel.nastasa@eli-np.ro (V.N.); paul.mereuta@nipne.ro (P.M.); keunhwan.kim@nipne.ro (K.K.); 4 Faculty of Physics, University of Bucharest, 030018 Bucharest, Romania; adriana.balan@unibuc.ro; 5 National Institute for Laser Plasma and Radiation Physics, Ilfov, 077125 Magurele, Romania; andra.dinache@inflpr.ro; 6 Doctoral School Materials Science and Engineering, National University of Science and Technology POLITEHNICA Bucharest, Splaiul Independentei No. 313, Sector 6, 060042 Bucharest, Romania; daniela.calin@stud.sefi.upb.ro; 7 Faculty of Physics, University of Bucharest Magurele, 077125 Magurele, Romania

**Keywords:** mollusk shells, calcium carbonate, calcium oxide, applications

## Abstract

Marine mollusk shells are a promising renewable source of calcium-based materials, offering a sustainable alternative for their synthesis. In this study, five types of marine shells—*Chamelea gallina*, *Mya arenaria*, *Rapana venosa*, *Mytilus edulis*, and *Pecten maximus*—were calcined at 900 °C for 2 h. The resulting powders were characterized by XRD, FTIR, SEM, PSD, and zeta potential analyses. XRD confirmed the dominant presence of CaO, with residual calcite and portlandite. FTIR spectra supported these findings, indicating the decomposition of carbonate phases and the formation of Ca–O bonds. SEM imaging revealed species-specific microstructures ranging from lamellar and wrinkled textures to compact aggregates, while particle size distributions varied from 15 to 37 μm. Thermogravimetric analysis revealed a two-step decomposition process for all samples, with significant species-dependent differences in mass loss and conversion efficiency, highlighting the influence of biogenic origin on the thermal stability and CaO yield of the resulting powders. Zeta potential measurements showed low colloidal stability, with the best performance found in *Rapana venosa* and *Pecten maximus* calcinated samples. Antibacterial activity was evaluated using a direct contact method against *Escherichia coli* and *Enterococcus faecalis*. All samples exhibited complete inactivation of *E. coli*, regardless of exposure time, while E. faecalis required prolonged contact (3.3 h) for full inhibition. The results highlight the potential of biogenic CaCO_3_ and CaO powders as functional, antimicrobial materials suitable for environmental and biomedical applications. This study underscores the viability of marine shell waste valorization within a circular economy framework.

## 1. Introduction

Calcium carbonate (CaCO_3_) and calcium oxide (CaO) are two essential inorganic compounds, closely linked by a thermal transformation relationship, in which CaCO_3_ acts as a direct precursor to CaO.

Both materials are found in common applications, due to their alkaline, neutralizing, and stabilizing character. Table 1 summarizes CaCO_3_ and CaO properties.

Also, these compounds are used in the building materials industry (manufacture of cement, mortar, and lime), in the treatment of acidic waters, in agriculture for soil pH correction, as well as in industry for the capture of acid gases (SO_2_, CO_2_) or as a flux in metallurgy [1,2,3]. In addition, CaCO_3_ has specific applications in the food, pharmaceutical, and cosmetic industries, as an additive or excipient, and CaO is valuable as a reagent in inorganic syntheses, purifications, and catalytic processes.

Common properties that determine the versatility of both compounds include reactivity with acids, neutralization ability, high-temperature stability, and natural abundance or affordability. Functionally, CaCO_3_ and CaO can be integrated into renewable chemical conversion cycles, with potential in carbon capture and reuse technologies, which gives them increased relevance in the context of sustainable development.

**Table 1:** CaCO_3_ and CaO properties.

Property	CaCO_3_ (Calcium Carbonate)	CaO (Calcium Oxide)	References
Aspect	White, crystalline or powder solid	White or gray solid, fine dust	[1,4]
Water solubility	A total of 0.013 g per 100 g of water at 25 °C	A total of 1 g/840 mL at 25 °C, 1 g/1740 mL at 100 °C	[5,6,7]
Acid reactivity	Forms salts and CO_2_	Forms salts and gives off heat	[1]
Alkaline properties	Weakly alkaline	Highly alkaline	[1,7,8,9]
Decomposition temperature	Breaks down at >825 °C	It is produced by the decomposition of CaCO_3_	[2]
Thermal behavior	At around 600–800 °C, it decomposes upon calcination into CaO and CO_2_	Highly thermally stable up to 2540 °C	[7]

Calcium carbonate (CaCO_3_) is a mineral that occurs in nature in several crystalline forms, each with specific characteristics, as follows: calcite (the most stable form, trigonal), aragonite (less stable, orthorhombic form), and vaterite (unstable, hexagonal). The crystalline form influences the physical and chemical properties of CaCO_3_. The extraction and processing of CaCO_3_ can have a negative impact on the environment through habitat destruction and carbon dioxide emissions. It can also be synthesized in the laboratory by various methods, each having advantages and disadvantages depending on the purpose of use. The most widely used methods are precipitation from aqueous solutions [7,8], calcium hydroxide carbonation, electrochemical synthesis, and the sol–gel method. The simplest and most used method is precipitation from aqueous solutions, where calcium chloride (CaCl_2_) reacts with sodium carbonate (Na_2_CO_3_), forming a precipitate of CaCO_3_.

CaCl_2_ + Na_2_CO_3_ → CaCO_3_↓ + 2NaCl (1)

Another effective method is calcium hydroxide carbonation (gas–liquid method), where carbon dioxide is passed through a suspension of Ca(OH)_2_, resulting in precipitation by CaCO_3_.

Ca(OH)_2_ + CO_2_ → CaCO_3_↓ + H_2_O (2)

This is an environmentally friendly method, as it can use CO_2_ resulting from other industrial processes. It also allows good control over the shape of the particles and ensures high purity of the product. However, it requires precise control of the rate of CO_2_ addition to avoid the formation of soluble calcium bicarbonate and may require a longer reaction time.

Calcium carbonate (CaCO_3_) can be synthesized through electrochemical approaches that couple ion generation with precipitation reactions. One common strategy involves the electrolysis of water, where hydroxide ions (OH^−^) formed at the cathode react with dissolved CO_2_ to generate carbonate ions (CO_3_^2−^), which subsequently precipitate as CaCO_3_ in the presence of calcium ions (e.g., from CaCl_2_ or Ca(NO_3_)_2_) [8]. Alternatively, direct electrochemical precipitation from electrolyte solutions containing both calcium and carbonate ions (e.g., via sodium bicarbonate) enables the formation of specific polymorphs, such as calcite or vaterite, depending on factors like ionic strength and the presence of additives (e.g., NaNO_3_) [9].

Electrocrystallization represents another route, whereby applied potentials drive the nucleation and growth of CaCO_3_ on electrode surfaces. This method is particularly relevant for scale control in industrial systems and can be tuned via electrode potential and dissolved oxygen concentration. Moreover, advanced techniques such as combined electrophoretic and electrochemical deposition have enabled the fabrication of hierarchical CaCO_3_ architectures, offering tailored surface morphologies with potential applications in catalysis and bone tissue engineering [10,11].

A more complex method, but with superior results in terms of fine particle quality, is the sol–gel method [12]. This involves the formation of a precursor gel, followed by a heat treatment, to obtain calcium carbonate. This technique allows the synthesis of homogeneous and fine particles and can be used to obtain compositions doped with other elements. Its disadvantages include process complexity, high costs, and the need for high temperatures for heat treatment.

Biogenic materials, such as mollusk shells or eggshells, are a sustainable source of calcium carbonate (CaCO_3_), with valuable potential in industrial, medical, and agricultural applications [13,14]. Unlike natural limestone, these biological resources are renewable, available in significant quantities as a by-product of the food industry, and can be processed to obtain high-purity materials. The technique for obtaining CaCO_3_ from shells is sustainable, capitalizing on biological waste and reducing dependence on limestone extraction. The advantages include high purity and biocompatibility. The disadvantages are collection and processing costs, variations in composition, and limited availability.

A brief comparative overview of biogenic and geological CaCO_3_ is presented in Table 2.

Compared to limestone extraction and chemical synthesis, obtaining CaCO_3_ from marine mollusk shells provides environmental benefits but requires process optimization.

CaO is mainly obtained by the calcination of limestone (CaCO_3_). The basic thermal process consists of the thermal decomposition of calcium carbonate at temperatures between 900 °C and 1100 °C, in a rotary or vertical furnace, according to the following reaction:

CaCO_3_ → CaO + CO_2_↑ (3)

This traditional method of processing is called “conventional calcination” and is the most widely used industrial technology. The efficiency of the process is influenced by the nature of the raw material (limestone purity), the granularity of the material, the temperature, and the calcination time in the oven.

Another more recent and energy-efficient method is concentrated solar-assisted calcination, in which heliostatic mirrors focus solar radiation to one point, generating high temperatures for the decomposition of limestone. This approach reduces CO_2_ emissions and fossil fuel consumption and is being intensively investigated in the context of the transition to more sustainable industrial processes [7]. Recently, research has also focused on the synthesis of calcium oxide at the nanoscale by wet chemical methods, such as coprecipitation [13], the sol–gel method [14,15], or the decomposition of organometallic precursors [7]. These methods allow the production of fine particles with a large specific area, useful in catalytic applications or in the field of advanced materials.

Unlike chemical, mechanical, or physical methods, the biogenic synthesis method uses natural biological resources that contain significant amounts of calcium in organic or inorganic forms, such as calcium carbonate (CaCO_3_) or calcium phosphates. Among the most used biogenic sources are mollusk shells (shells, sea snails), eggshells, animal bones, corals, or certain types of calcareous algae [16,17,18,19,20,21,22,23,24,25]. These resources are abundant, renewable, and often considered biological waste.

The synthesis process primarily involves the collection, cleaning, and drying of the biomass, followed by a mechanical pre-treatment (grinding) step to increase the specific surface area. Subsequently, the material is subjected to a controlled heat treatment, usually at temperatures between 700 °C and 1000 °C, depending on the nature of the source, to induce calcium compounds. The main reaction is like the conventional one:

CaCO_3_ → CaO + CO_2_ (4)

However, the advantage of biogenic synthesis lies in the presence of a porous microstructure and organic impurities that favor heat dispersion and the formation of calcium oxides with fine morphologies and increased reactivity. In many cases, the product obtained is in the form of nanoparticles or porous aggregates, with a large specific area, which is essential for applications in catalysis, biodiesel, environmental remediation, and biomedicine [19,20,22,24,25].

Biogenic synthesis brings significant advantages compared to traditional industrial methods. It recovers biological waste, requires relatively low energy consumption, reduces greenhouse gas emissions, and provides better control over the physicochemical properties of the final product [26,27]. Also, the use of biogenic sources allows the integration of this process into a circular economy, contributing to reducing the ecological impact of the chemical industry. A brief comparative overview of CaO synthesis methods is presented in Table 3.

The comparative analysis of chemical and biogenic synthesis methods highlights the fact that, although classical chemical methods—such as limestone calcination or sol–gel synthesis—offer products with high purity and structural control, they involve substantial energy consumption and significant ecological impact. Instead, biogenic synthesis offers a sustainable and efficient alternative, capitalizing on natural organic waste and providing CaO with porous morphology and increased reactivity, suitable for applications such as wastewater treatment, CO_2_ absorption, composite fabrication, and catalytic supports.

Also, nanostructured variants obtained by advanced methods have potential in emerging fields such as biotechnology, medicine (e.g., as bioactive bone material), and energy storage. The choice of the synthesis method must thus be correlated with the requirements of the targeted application, balancing the costs, the environmental impact, and the functional properties of the resulting material.

Recent studies have focused on their antibacterial properties, which are of relevance in the context of developing novel, eco-friendly antimicrobial materials for biomedical and environmental applications. In its bulk form, CaCO_3_ is biologically inert and does not exhibit significant antibacterial activity. However, its nanostructured derivatives have shown promising results, particularly when functionalized or doped with antimicrobial agents such as silver (Ag^+^), zinc (Zn^2+^), or copper (Cu^2+^) ions. In these hybrid systems, CaCO_3_ primarily acts as a carrier matrix, enabling the sustained release of active species and promoting enhanced interaction with bacterial membranes due to its high surface area and porosity [29].

Studies have demonstrated that silver-doped CaCO_3_ nanoparticles can inhibit the growth of both Gram-positive and Gram-negative bacteria, including Staphylococcus aureus and *Escherichia coli*. The antibacterial effect is largely attributed to the slow ion release and localized toxicity induced by the metal ions, while the CaCO_3_ matrix ensures biocompatibility and structural stability [30,31].

Siek et al. [32] investigated tricalcium phosphate α-TCP-based bone cements modified with silver-doped hydroxyapatite and calcium carbonate. Calcium compounds (including CaCO_3_ and CaO) contribute to indirect antibacterial activity by increasing local pH and promoting the formation of antibacterial bioactive phases (such as Ag+).

Calcium hydroxide (Ca(OH)_2_) is commonly employed as a temporary intracanal medicament in endodontics due to its strong antimicrobial activity, on Enterococcus facaelis film, effectively preventing bacterial regrowth within the root canal system between treatment sessions [32,33]. In the meantime, upon contact with water, CaO reacts to form calcium hydroxide (Ca(OH)_2_), significantly raising the pH of the surrounding environment (typically >12) [31]. This alkaline environment disrupts bacterial cell walls and denatures essential proteins, leading to rapid microbial inactivation. Biofilms exposed to Ca(OH)_2_ showed increased Ca^2+^ uptake due to the alkaline pH, which in turn altered the physicochemical characteristics of the *E. faecalis* biofilm, as mentioned by [32,33,34,35] Jimenez-Gonzalez et al. mentioned the properties of Ca(OH)_2_-based sealants, including biocompatibility, antibacterial activity, sealing ability, and the promotion of periapical healing. Its use as intracanal medication is mainly attributed to its antimicrobial effect and its ability to stimulate tissue repair [35]. Mohammadi et al. [33] and Arandi [36] emphasized the wide use of Ca(OH)_2_ in endodontic treatments, due to its broad spectrum of antibacterial activity, its ability to neutralize endotoxins, and its relative biocompatibility with other antiseptics. Owing to this multi-mechanistic action, CaO has been found to be effective against a broad spectrum of pathogenic bacteria and is currently being explored for applications in disinfection, waste treatment, and antibacterial construction materials.

In addition to its high pH effect, CaCO_3_ and CaO may contribute to the formation of reactive oxygen species (ROS), which further damage bacterial DNA and cellular components [36,37].

The novelty of this study lies in its comparative approach using five distinct species of marine mollusk shells (*Chamelea gallina*, *Mya arenaria*, *Rapana venosa*, *Mytilus edulis*, and *Pecten maximus*) as precursors for CaO powders. Unlike previous reports that focus on a single source, our work systematically correlates the biogenic origin, phase composition, morphology, and colloidal behavior with antibacterial efficacy, highlighting species-specific functionalities. The present work aims to comparatively characterize the crystalline phases, morphology, particle size, surface charge, and antibacterial performance of the resulting powders following calcination. There is a lack of comprehensive, comparative studies evaluating how different mollusk-derived Ca-based materials vary in structure, reactivity, and functional performance. Most literature treats bio-Ca-based materials generically, without addressing the influence of precursor species on microstructure and antibacterial behavior. This limits material optimization for targeted applications.

The results offer insights into how biogenic origin and processing conditions influence the functional properties of Ca-based materials and underscore their potential in sustainable and antimicrobial applications. Our findings contribute to the field by providing a framework for tailoring CaO properties via precursor selection. By demonstrating that specific shells (e.g., *Rapana venosa* and *Pecten maximus*) yield powders with higher reactivity and antibacterial performance, we offer practical insights for the design of sustainable, application-specific CaO materials, particularly in water treatment, biocomposites, and environmental remediation.

## 2. Materials and Methods

Marine mollusk shells (*Chamelea gallina*, *Mya arenaria*, *Rapana venosa*, and *Mytilus edulis*) were collected from the Black Sea shore. Only scallops, *Pecten maximus* (Saint Jaques shells), were bought from the fish market. All shells were rigorously washed for the removal of organic matter, dried, ground, and washed again with demineralized water. From each type of shell, 20 g was subjected to a calcination process at 900 °C to remove residual organic compounds and convert them into CaCO_3_ or CaO. For calcination, a Mikrotest MKF-05, Ankara, Türkiye, electric muffle oven was used. During this period, the furnace operated for 43.75 min continuously at nominal power (2 kW), since it must supply energy to increase the temperature from 25 °C to 900 °C with 20 °C/min. The energy consumed for heating was 1.458 KWh (2 kw × 43.75 min). In the maintenance stage, the oven worked intermittently (through the thermostat); however, as a conservative estimate, we consider that it operated on average at 50% of the rated power (to maintain the temperature). After the oven had reached 900 °C, it only needed to compensate for heat loss, resulting in a consumption below 50%. If we consider that energy consumption in the maintenance stage for 2 h was 50%, the energy consumed was 2.0 kWh. For a controlled cooling rate, we estimate 30% of the nominal power during 43.75 min, meaning an energy consumption of 0.43 kWh. Therefore, the total energy consumption was 3.8955 kWh. The average electricity tariff in The European Union, in 2024, was EUR 28.9/100 kWh, which means EUR 1.164. Taking into account that all 5 samples are from natural resources and were calcined at the same time, the energy cost per sample was EUR 0.2328.

After calcining, the powder obtained was weighed again, and the gravimetric calcination yield was determined. Considering that the theoretical efficiency of transforming CaCO_3_ into CaO was 56.08%, and considering that after calcination the complete transformation into CaCO_3_ would be achieved, we also calculated the theoretical efficiency of total transformation into CaO. The characteristics of the powders are shown in Table 4.

The gravimetric yields obtained varied between 54.3% and 64.9%, indicating good efficiency of the conversion process, but also a notable variation between species. The highest yield value was recorded for the Saint Jacques species (64.93%), which suggests a higher CaCO_3_ content in the shell structure or better thermal efficiency in the calcination process. On the other hand, species such as *Chamelea gallina*, *Mya arenaria*, and *Mytilus edulis* generated yields of around 54–55%, which may indicate either a more porous structure with higher mass losses (including organic matter or impurities) or a lower initial CaCO_3_ content. In practical terms, yields above 50% are considered good for the biogenic synthesis of CaO, especially considering that the materials used are free natural residues or have low economic value. Therefore, these results support the viability of the biogenic synthesis of CaO as an environmentally friendly and efficient method for the recovery of marine litter, with application potential in water treatment, composite materials, or ecological fertilizers. Variations in calcination yield can be attributed to biological and structural differences in shells, such as wall thickness, proportion of organic compounds, and type of CaCO_3_ (calcite, aragonite) crystallinity.

In this context, the structural, compositional, and morphological analysis of these calcined powders becomes essential for assessing the quality and areas of applicability.

The structural characterization of the material was performed using X-ray diffraction (XRD) in Bragg–Brentano geometry (θ–2θ), operating in reflection mode. Measurements were carried out using a copper (Cu) anode X-ray tube, emitting characteristic radiation with a Kα_1_ wavelength of 1.5406 Å. The operating conditions of the generator were set to 40 kV and 40 mA. Data were collected over a 2θ range of 10° to 70°, with a step size of 0.02° and an acquisition time of approximately 3 s per point. Signal detection was achieved using a scintillation detector operated at a high voltage of 615 V and an amplifier gain of 80. The resulting diffraction patterns were analyzed to identify the crystalline phases by comparing the peak positions and intensities with reference data from the ICDD database.

Fourier transform infrared (FTIR) spectroscopy with attenuated total reflectance (ATR) was employed to identify the main functional groups present in the synthesized calcium-based materials. The spectra were recorded in the range of 4000–350 cm^−1^ using a Bruker Tensor 27 spectrometer (BrukerOptik GmbH, Ettlingen, Germany) equipped with a diamond ATR accessory, at a spectral resolution of 4 cm^−1^, and averaging 32 scans per sample.

Thermogravimetric analysis (TGA/SDTA Mettler Toledo, Greifensee, Switzerland) was performed in an 80 mL/min nitrogen flow at a heating rate of 10 °C/min in the temperature range of 25–1000 °C. The 5–15 mg samples were placed in 80 µL alumina crucibles. Data were collected and interpreted using Star 9.10 software (Mettler Toledo, Greifensee, Switzerland), while graphs were plotted in Origin 2023b (OriginLab Corporation, Northampton, MA, USA).

The surface morphology and microstructural features of the synthesized calcium-based materials were investigated using scanning electron microscopy (SEM) with an SU 8230 Microscope (Hitachi High-Tech Corporation Hitachinaka, Ibaraki, Japan). The SEM images were acquired with a high-vacuum field emission gun (FEG) microscope operated at an accelerating voltage of 5.0 kV, with a working distance of 4.5 mm. Both low-magnification (×1000) and high-magnification (×30,000) images were recorded to provide an overview of the particle aggregation, as well as fine surface features.

Particle size distribution (PSD) measurements were conducted using a Partica mini-LA-350 Laser Scattering Particle Size Distribution Analyzer (Horiba Ltd., Kyoto, Japan). This analytical instrument employs the laser diffraction technique, a well-established method based on the angular variation in intensity of light scattered as a laser beam passes through a dispersed particulate sample. The samples were dispersed in distilled water to ensure uniform suspension and prevent agglomeration. The dispersion process was optimized to maintain particle integrity and avoid size alteration. All measurements were performed in accordance with the manufacturer’s standard operating procedures to ensure the consistency and reproducibility of the results. Zeta potential measurements were carried out using a Horiba SZ-100V2 Nanoparticle Analyzer (Horiba Ltd., Kyoto, Japan), which utilizes electrophoretic light scattering (ELS) to determine the electrokinetic potential of particles in suspension. This technique measures the velocity of charged particles under an applied electric field, providing insight into the surface charge characteristics and colloidal stability of the sample. Samples were prepared by dispersing the particles in distilled water, ensuring adequate dilution and homogenization to minimize multiple scattering effects and particle interactions. Solutions were prepared at a concentration of 1 mg/mL in distilled water. They were ultrasonicated for 1 h before the pH measurements were performed. The pH was measured with a Lab 860 pH meter (Schott Instruments, Wolverhampton, UK).

To investigate the antibacterial properties of calcinated mollusk shells against *Escherichia coli* and *Enterococcus faecalis*, a direct contact approach was used. The following reference strains (qualitative, with an unknown number) were used: *Escherichia coli* ATCC 25922 and *Enterococcus faecalis* ATCC 19433 from IeLab (American Type Culture Collection, ATCC). To determine the antibacterial activity, the colony counting method and the membrane filtration technique were used, according to [29]. Each loop inoculated with bacterial load was hydrated in 1 mL of sterile distilled water for 30 min at 37 °C. Subsequently, using a sterile loop, inoculations were performed on a non-selective medium, Tryptic Soy Agar, followed by incubation at 37 °C for 24 h. From these subcultures, working cultures were prepared using the same procedure. Bacterial suspensions were then collected from the working cultures and transferred into 10 mL of sterile distilled water.

To estimate the approximate number of bacteria, a McFarland densitometer was used. Considering that a 0.5 McFarland standard corresponds to a bacterial concentration of approximately 1.5 × 10^8^ CFU/mL, the initial values recorded were 0.4 McFarland for *Enterococcus faecalis* and 0.2 McFarland for *Escherichia coli*. Since readings below 0.5 McFarland may involve measurement errors, serial decimal dilutions were performed up to 10^−4^ for *Escherichia coli* and 10^−3^ for *Enterococcus faecalis*. Each dilution was subsequently tested to determine the number of viable bacteria, particularly the count of visible colonies on the culture media.

The selective culture media used were Merck Chromocult Coliform Agar and Slanetz and Bartley Agar, prepared according to the manufacturer’s instructions, at 41.5 g/L for Slanetz–Bartley Agar and 26.5 g/L for Chromocult Coliform Agar.

The experimental design aimed to evaluate the extent of bacterial viability as a function of contact time with the test material. For each test, 0.01 g of sample powder was used, previously sterilized under UV light for 15 min to prevent initial contamination. Bacterial suspensions were prepared, and artificial test samples were obtained by mixing 9 mL of sterile distilled water with 1 mL of the respective bacterial suspension. The initial bacterial loads were approximately 1.5 × 10^5^ colony-forming units (CFU) for *Escherichia coli* and 5 × 10^5^ CFU for *Enterococcus faecalis*. The positive control (M^+^) contained only sterile distilled water and bacterial suspension, while the negative control (M^−^) included sterile distilled water only.

The calcium-based powders were immersed in the bacterial suspensions, and two experimental conditions were assessed: (i) short contact time (<30 min), followed by immediate plating on selective culture media (Chromocult Coliform Agar for *E. coli* and Slanetz and Bartley Agar for *E. faecalis*); and (ii) extended contact time (3.3 h) at room temperature (25 °C), followed by incubation at 37 °C for 24 h.

All measurements were performed under ambient conditions, adhering strictly to the manufacturer’s standard operating procedures to maintain consistency, reliability, and reproducibility of the data.

## 3. Results

### 3.1. Structural Analysis

According to JCPDS–International Centre for Diffraction Data, Newtown Square, PA, USA, the diffraction angles of CaO (calcium oxide), PDF No. 37-1497, 2001; CaCO_3_ (calcite), PDF No. 05-0586, 2001; CaCO_3_ (aragonite), PDF No. 01-0628, 2001; CaCO_3_ (vaterite), PDF No. 33-0268, 2001; and Ca(OH)_2_ (portlandite) PDF No. 01-073-5492 are presented in Table 5.

**Figure 1:**
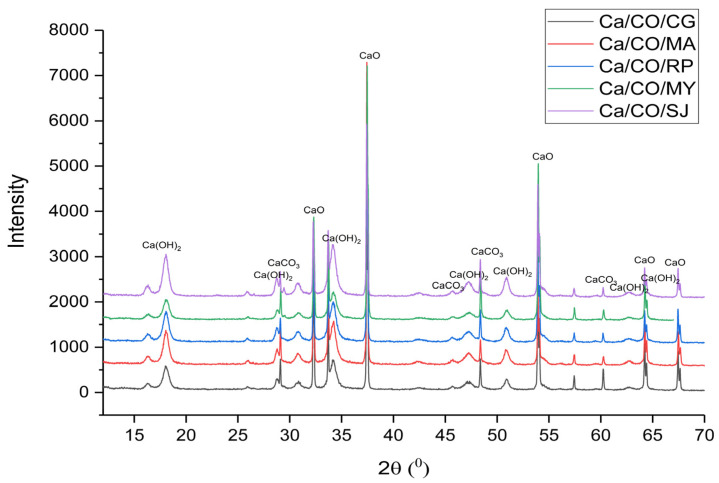
XRD patterns of calcinated shells.

The structural analysis of the calcium-based materials was performed using X-ray diffraction (XRD), with the diffraction patterns shown in Figure 1. The corresponding diffraction data, including the peak positions and identified phases, are summarized in Table 5.

For the Ca/CO/CG sample, the XRD pattern revealed dominant peaks associated with lime (32.35°, 37.44°, 54.00°, 64.37°, and 67.33°), with additional signals from calcite (28.74°, 47.3°, and 60.32°) and portlandite (18.11°, 34.32°, 47.36°, 51.02°, 62.87°, and 64.49°). Similarly, the Ca/CO/MA, Ca/CO/RP, Ca/CO/MY, and Ca/CO/SJ samples displayed clear evidence of CaO as the primary crystalline phase, complemented by varying degrees of calcite and portlandite presence.

These findings indicate that, during the calcination process at 900 °C, the primary reaction is the thermal decomposition of CaCO_3_ into CaO and CO_2_. The presence of residual calcite in some samples suggests that complete conversion was not achieved, possibly due to variations in particle size, heating uniformity, or intrinsic material properties. Moreover, the identification of portlandite in the XRD patterns points to the post-calcination hydration of CaO when exposed to ambient moisture.

The XRD analysis confirms the successful transformation of the shell materials into high-lime content powders, suitable for applications where reactive CaO is desired. However, the residual phases highlight the importance of optimizing the calcination parameters to ensure complete transformation and to control the final material composition for specific industrial or environmental uses.

### 3.2. Compositional Analysis by Spectroscopy

**Figure 2:**
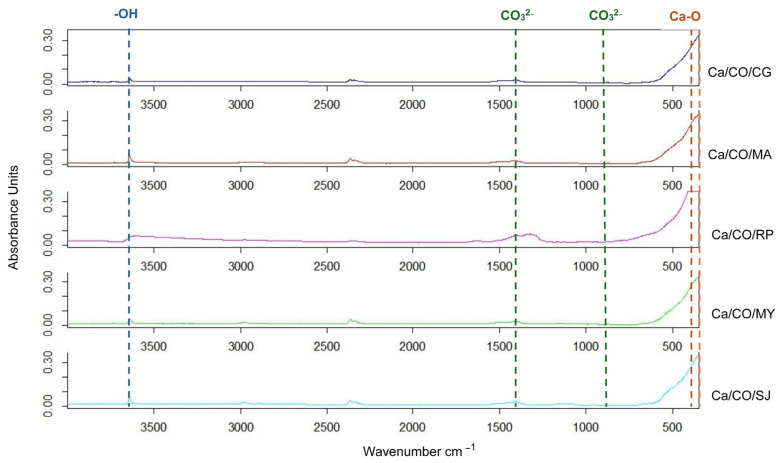
ATR-FTIR spectra of calcinated shells.

In this study, the FTIR spectra of calcium-based materials obtained after calcination at 900 °C for 2 h were analyzed. The spectra were recorded in the range of 4000–350 cm^−1^, covering the main absorption regions relevant for the identification of the chemical composition of the samples, as presented in Figure 2.

The spectra obtained have the following characteristics:

○ In the region of 3500–3000 cm^−1^, associated with O–H (hydroxyl or hydration water) vibrations, signals are very weak or absent, indicating complete removal of structural water by heat treatment.
○ In the 2500–1500 cm^−1^ area, where bands specific to the C=O or C–O groups would normally be observed, the spectra are almost flat, suggesting the almost complete elimination of organic matter from the composition of the shells following calcination.
○ In the imprint region, 1500–500 cm^−1^, the spectra show a significant increase in absorption, especially below 600 cm^−1^. This corresponds to the Ca–O vibrations characteristic of calcium oxide (CaO), confirming the decomposition of CaCO_3_ (calcium carbonate) into CaO by the calcination reaction.

Based on the interpretation of the spectra, we can identify the following main phases, listed in Table 6:

CaCO_3_ (calcium carbonate), with characteristic bands around ~1400, with ~870 corresponding to the vibrations of the CO_3_^2−^ group.

CaO (calcium oxide), identified by bands in the ~500–350 cm^−1^ range, generated by Ca–O vibrations.

Ca(OH)_2_ (calcium hydroxide), justified by post-calcination moisture, evidenced by a weak band in the area of ~3640 cm^−1^ (O–H stretching).

### 3.3. Dimensional Analysis

**Figure 3:**
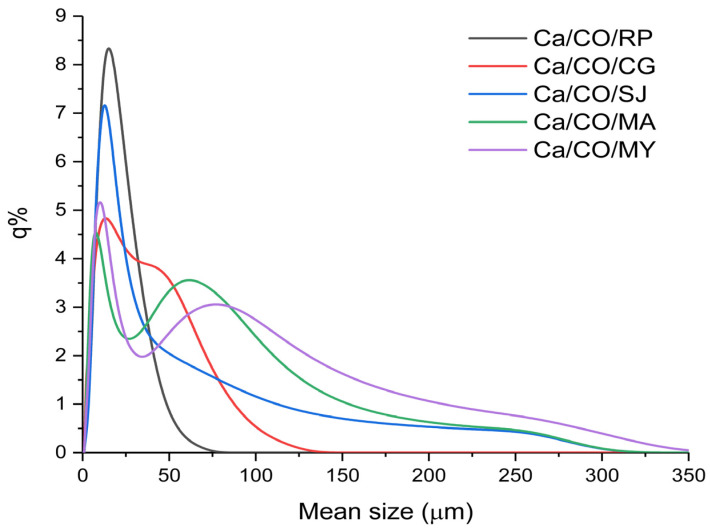
Percentage frequency of mean size.

In this analysis, the five samples of shells calcined at 900 °C for 2 h were investigated. The mean size is presented in Table 7. The graph in Figure 3 shows the percentage frequency (q%) of particles for each size class.

Ca/CO/CG powder (red curve) has an extremely wide and dispersed distribution, with a notable presence of large and small particles. It is less homogeneous but can be useful in applications where this particle size variation confers an advantage, such as composites or filler applications. The Ca/CO/MA powder size distribution (represented by the green curve in the graph) shows a specific profile, characterized by a wide range of particle sizes. The main peak of the distribution is located around 70–80 μm, which indicates the predominant particle size in the sample. The presence of both fine and coarse particles, evenly distributed over the range of 50–150 μm, reflects a balanced mixture that can influence the final properties of the resulting material. Such a distribution can help to reduce the total specific surface area, however, at the same time, it can improve characteristics such as permeability, porosity, and mechanical behavior in specific applications (e.g., as filler or in composites).

The Ca/CO/RP powder (gray curve) has a monomodal distribution with an initial maximum at fine particles (~25 μm) and an extension to medium sizes (~75 μm). This indicates a combination of fineness and robustness, suitable for structural applications or special mortars. The distribution of Ca/CO/MY powder (purple color) is relatively wide, with a maximum in the medium particle area (~20 μm) and tail extended to 200 μm. The sample is versatile, suitable for both reactive applications and porous solid media. The wide and asymmetrical distribution (blue curve) of the particles in the Ca/CO/SJ powder, with expansion towards values above 150 μm, suggests a polydisperse material, with the presence of large particles. It is suitable for applications in which large particle size favors permeability, such as water treatment or filtration.

### 3.4. Thermal Behavior

**Figure 4:**
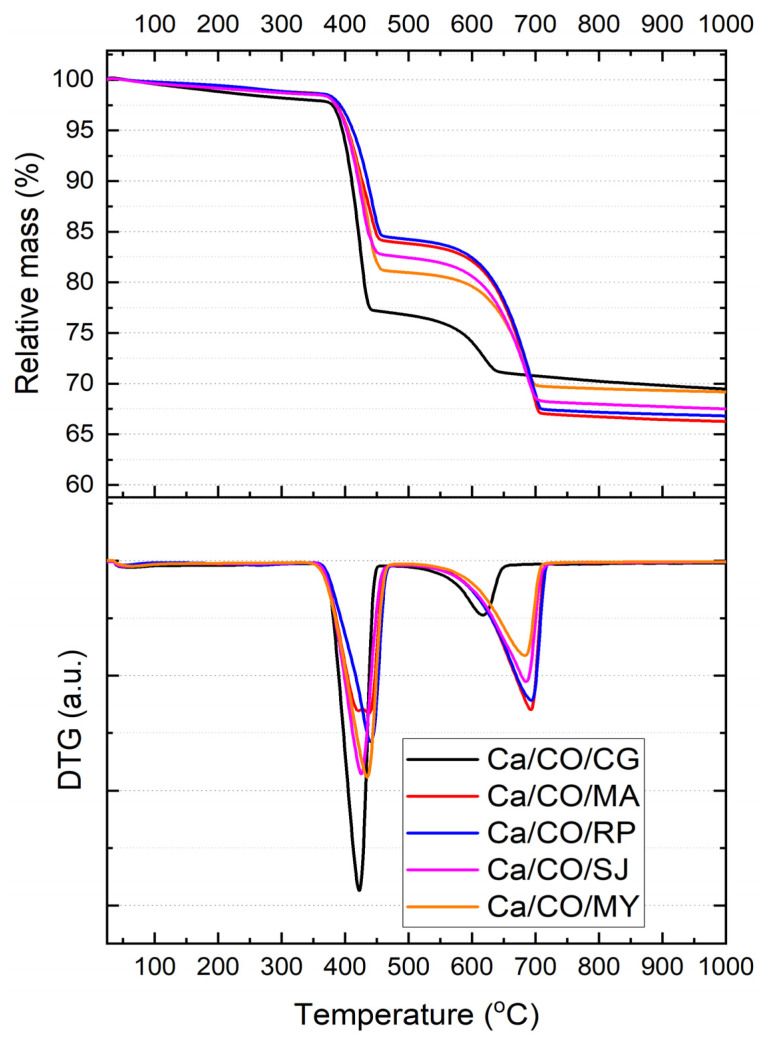
TGA thermograms of Ca/CO samples in 80 mL/min nitrogen flow at a heating rate of 10 °C/min and the corresponding first-order derivatives of the TGA curves showing the maximum reaction rate points.

Figure 4 presents the thermogravimetric analysis (TGA) curves and their corresponding first derivatives (DTG) for the five calcium-based powders obtained by calcination of marine mollusk shells in nitrogen atmosphere at a heating rate of 10 °C/min. All samples exhibit a two-step mass loss profile, corresponding to distinct thermal decomposition events. The key thermal parameters extracted from the TGA thermograms are summarized in Table 8.

The first decomposition step, associated with the release of physically adsorbed water and partial degradation of organic matter, occurs between ~388 and ~437 °C. The corresponding mass loss (Δm_1_) ranges from 15.85% (Ca/CO/RP) to 23.18% (Ca/CO/CG), with Ca/CO/CG exhibiting the greatest weight loss and the sharpest DTG peak (T_max,1_ = 418.43 °C). This may be attributed to the presence of more labile carbonate phases (calcite according to XRD). The onset temperature of this event is highly consistent across all samples (~388–395 °C), suggesting similar early-stage decomposition mechanisms, regardless of species.

The second decomposition event, spanning 615–696 °C, corresponds to the complete decarbonation of residual CaCO_3_ and transformation into CaO. The samples derived from *Mya arenaria* (Ca/CO/MA) and *Rapana venosa* (Ca/CO/RP) show the highest Δm_2_ values of 17.13% and 17.02%, respectively, indicating a significant residual carbonate fraction after the first step. Conversely, Ca/CO/CG shows a markedly lower Δm_2_ (6.44%), implying a more complete early decomposition or reduced carbonate content in the original material.

The residual mass at 1000 °C (m_residuu_) ranged from 66.28% (Ca/CO/MA) to 69.49% (Ca/CO/CG). The highest residue values (CG and MY) suggest either higher CaO content or incomplete decomposition of carbonate material. These results are consistent with XRD findings, which indicate residual calcite and portlandite phases in Ca/CO/CG and Ca/CO/MY.

### 3.5. Morphological Analysis

**Figure 5:**
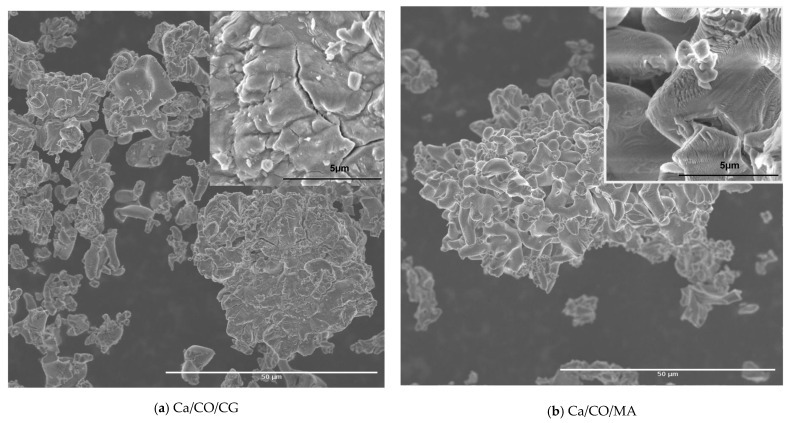

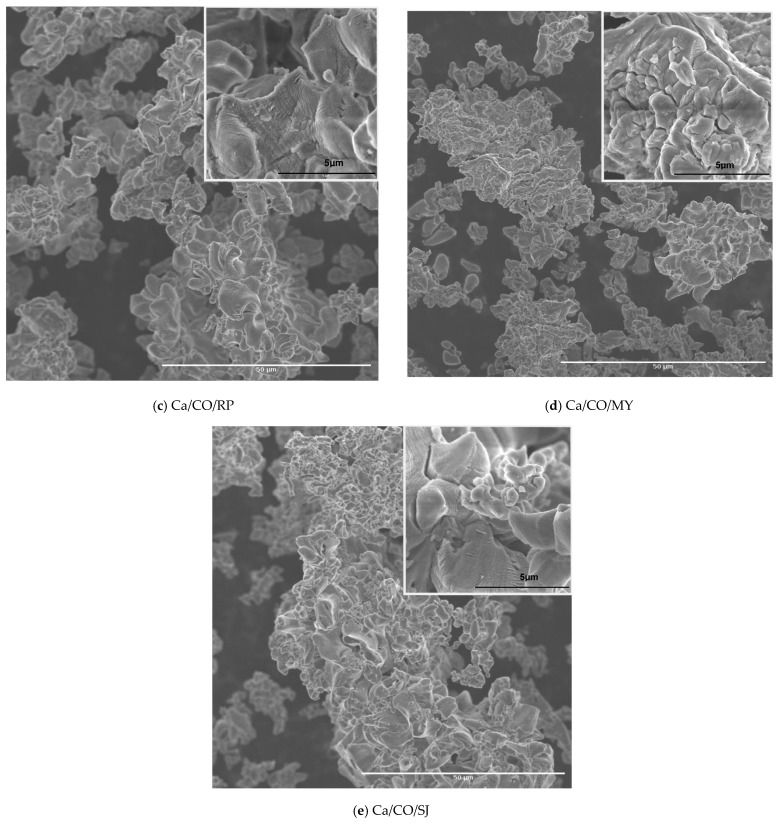
SEM micrographs of: (**a**) CaCO_3_/CG; (**b**) CaCO_3_/MA; (**c**) CaCO_3_/RP; (**d**) CaCO_3_/MY; (**e**) CaCO_3_/SJ.

The surface morphology and microstructural features of the synthesized calcium-based powders investigated by SEM were recorded to provide particle aggregation, as well as fine textures, as shown in Figure 5.

The SEM image shown in Figure 5a highlights the morphology of the powder resulting from the calcination process of *Chamelea gallina* shells at different magnifications. At a magnification of 1000×, an aggregate microstructure is observed, made up of particles with morphology formed by randomly distributed thin and wrinkled sheets. The insertion at 10,000× reveals fine topographic details, including lamellar edges and wavy particle texture, typical characteristics for structures resulting from the thermal disintegration of carbonate components. The morphology obtained suggests a significant porosity and good surface accessibility, essential features for applications in reactive or ion exchange systems.

The SEM image in Figure 5b highlights the structure of the powder resulting from the calcination of the shells of *Mya arenaria*, showing compact aggregates of particles with laminated morphology and irregular edges. At a magnification of 1000×, formations characterized by dense agglomerations and a relatively homogeneous distribution on the examined area are observed. The detail at 10,000× reveals fine details of the particles, such as wavy textures and microfractures, indicating a brittle structure and partial peeling of the sheets. This morphology suggests an extended specific surface area and moderate porosity, favorable aspects for uses in catalytic, sorption applications or as a precursor material for advanced syntheses. The resulting structure reflects the transformations undergone by the carbonate compounds during heat treatment, leading to the formation of reactive solid networks.

The SEM image in Figure 5c highlights the micro- and nanoscopic structure of the powder obtained by the calcination of *Rapana venosa* shells. At 1000× magnification, the particles have a well-defined flower-like morphology, consisting of thin, slightly twisted sheets with a layered texture, like wrinkled petals. These aggregates exhibit good dispersion and significant porosity. The topographic details visualized in the 10,000× insert highlight the roughness and partial exfoliation of the layers, confirming the fragile nature of the network formed by thermal disintegrations. This hierarchical structuring resulting from calcination reflects an internal reorganization of the shell-specific carbonate compounds, providing a material with high potential in the engineering of the environment or porous materials. After the calcination of the shells of *Mytilus edulis*, in Figure 5d, irregular aggregates formed by fragmented and laminated particles are indicated. At a magnification of 1000×, a heterogeneous distribution of particles is observed, with a tendency to agglomerate in compact formations and a finely granular texture. The detailed image at 10,000× reveals a fractured-looking surface with multiple microcracks, with pronounced relief and sharp edges, a sign of heat stress and structural disintegration during calcination. The observed texture reflects the complex composition of the Mytilus edulis shell, rich in calcium carbonate and organic components, susceptible to intense morphological transformations under heat treatment.

The SEM image of the powder obtained by calcination of the *Pecten maximus* shells in Figure 5e, obtained at a magnification of 1000×, highlights a complex morphology, characterized by folded particles and well-contoured lamellar edges, forming porous aggregates with good spatial dispersion. The surface of these particles has a wavy texture and successive layers, features specific to structures formed by the gradual calcination of carbonate materials. The detailed image obtained at 30,000× provides a glimpse into the microstructure. The presence of smooth and continuous surfaces, furrowed by fine faults and regular undulations, is noted, indicating a controlled thermal transformation and a relatively compact structure, compared to the other species analyzed. This morphology denotes a lower porosity, but a higher structural strength, being suitable for applications where materials with a stable surface are required, such as in ceramic syntheses, bio composites, or support materials in catalytic processes.

One limitation of this study lies in the absence of specific surface area measurements (e.g., BET analysis) and pore size distribution data. While SEM imaging allowed qualitative insight into particle morphology, the lack of quantitative surface area characterization limits the depth of structural analysis.

### 3.6. Zeta Potential Analysis

Each sample was dispersed in distilled water and placed in a measuring tank equipped with integrated electrodes, where a constant electrical voltage of 2.3 V was applied to generate a uniform electric field. The charged particles moved under the influence of this field, and their movement was monitored by coherent light scattering (ELS). The experimental parameters were carefully controlled to ensure the reproducibility of the results. The average temperature of the measuring cell was maintained in the range of 24.8–25.0 °C, and the viscosity of the dispersion medium (distilled water) was between 0.894 and 0.985 mPa·s. The conductivity of the medium varied between 5.606 and 6.673 mS/cm. The pH of all solutions is >12.50, according to Table 9.

Zeta potential analysis reveals the colloidal stability of the suspensions of obtained calcium-based powders. The samples with the lowest zeta potential, such as Ca/CO/MA (−0.2–0.8 mV), indicate a high tendency for aggregation and sedimentation, being poorly stable in suspension. On the other hand, Ca/CO/RP (3.6–13.3 mV) and Ca/CO/SJ (0.9–14.5 mV) have moderate values, but still below the threshold of ±30 mV, considered optimal for stable colloidal suspensions. The Ca/CO/CG (1.8–3.6 mV) and Ca/CO/MY (1.9–2.2 mV) samples complete the spectrum with low values, indicating a poorly stable dispersion. However, none of the samples exceeded the threshold considered optimal (>±30 mV) for stable colloidal suspensions.

All of the samples analyzed show low values of zeta potential, mostly below the threshold of ±10 mV, which indicates the following:

- Unstable or poorly stable suspensions.
- Pronounced tendency for aggregation, agglomeration, and sedimentation in the absence of a stabilizer.
- The need for optimizations (e.g., pH adjustment, surfactant addition, and ultrasonic dispersion) to improve colloidal dispersion for applications.

Correlating the datasets from Table 8, we notice that there is no direct relationship between particle size and zeta potential. The Ca/CO/CG sample showed an average particle size of 20.1 μm and an average zeta potential of 2.5 mV, indicating very low colloidal stability. Similarly, Ca/CO/MA, with a larger size (31.9 μm), recorded an average zeta potential of only 0.2 mV, being in the vicinity of the isoelectric point, where the net surface charge is minimal and the risk of coagulation is at maximum.

The Ca/CO/RP sample showed an average size of 15.1 μm and recorded the highest values of zeta potential among all of the samples analyzed, with a peak of 13.3 mV and an average value of about 7.9 mV. Although these values do not fall within the range of strong colloidal stability, they indicate a significantly higher degree of electrostatic repulsion compared to the other samples, suggesting moderate colloidal stability.

In the case of the Ca/CO/MY suspension, with a mean size of 36.9 μm, the zeta potential ranged between 1.9 and 2.2 mV, indicating an unstable colloidal state, like Ca/CO/CG and Ca/CO/MA. In contrast, Ca/CO/SJ showed considerable variability between the three measurements (from 0.9 mV to 14.5 mV), suggesting either a heterogeneous distribution of charges per particle or a temporal evolution of suspension aggregation.

It is notable that all samples show values of the zeta potential below the threshold of 15 mV, which reflects limited colloidal stability under the conditions tested.

Although all calcium-based suspensions exhibited a strongly alkaline environment (pH ≈ 12.7), the measured zeta potential values remained relatively low across all samples (<15 mV), indicating limited electrostatic stabilization. This apparent decoupling between high pH and low zeta potential suggests that, despite the presence of excess OH^−^ ions in the solution, the surface of the CaO/Ca(OH)_2_ particles does not develop a significant surface charge density under the tested conditions. The observed variability in zeta potential—highest in Ca/CO/RP and Ca/CO/SJ—can be attributed to subtle differences in surface morphology and phase composition, which may affect the degree of hydroxide ion adsorption or Ca^2+^ release at the solid–liquid interface. Furthermore, the proximity of the system to the isoelectric point (IEP) of calcium hydroxide (~pH 12.4–12.6) may partially explain the low net surface charge and the tendency toward aggregation observed in all samples. Ca/CO/RP combines fine particles with relatively good zeta potential, making it a promising candidate for colloidal applications, while Ca/CO/MY, although it has large particles, exhibits low suspension stability. This observation emphasizes the importance of chemical composition, surface morphology, and post-calcination treatments in determining final colloidal properties.

In conclusion, for applications requiring stable dispersions in water, Ca/CO/RP and Ca/CO/SJ samples could be optimized by adjusting the suspension parameters (pH and surfactants), while, for solid applications (catalysis, composites, and filtration), the selection will be made according to the desired fineness and physicochemical properties of the particles. This analysis demonstrates the high potential of biogenic synthesis from marine shells to obtain sustainable functional materials.

### 3.7. Antibacterial Activity Analysis

The anterior studies [30,31,32,33,34,35,36,37] clearly demonstrate that calcium-based materials, especially CaO, Ca(OH)_2_, and CaCO_3_, exhibit notable antibacterial properties, giving them considerable potential for biomedical and dental applications. The antibacterial activity of these compounds derives mainly from their ability to induce a high pH environment, destabilizing bacterial cell membranes and affecting their viability [30,31,32,33,34,35,36,37]. In particular, the ecologically synthesized CaCO_3_ nanoparticles from eggshells showed a remarkable antibacterial and antibiofilm effect, opening perspectives for the recovery of biogenic waste for therapeutic purposes. Also, the synergy between CaO or Ca(OH)_2_ and antimicrobial agents, such as silver nanoparticles, or superoxidized solutions, has led to improved antibacterial efficacy against Gram-positive and Gram-negative bacteria, including *Enterococcus faecalis*, a species recognized for its resistance in endodontic infections. Thus, calcium-based compounds represent a versatile class of biomaterials with relevant antimicrobial functions, being particularly promising in the field of nanomedicine, minimally invasive dental treatments, and bone regeneration.

The data in Table 10 summarize the antibacterial activity of CaCO3, Ca(OH)_2_, and CaO according to [30,31,32,33,34,35,36,37].

In our study, an evaluation *of E. faecalis* and *E. coli* was carried out after 30 min and 3.3 h of being exposed to Ca-based materials elaborated from mollusk shells. Quantitative analysis of 1 mL aliquots from each suspension, containing approximately 500 CFU of *E. faecalis* and 100 CFU/mL of *E. coli*, revealed the following, as presented in Table 9:

○ Complete inactivation of *Escherichia coli* was observed in all tested samples, regardless of the contact time.
○ *Enterococcus faecalis* exhibited complete inhibition only in samples subjected to a prolonged contact time of 3.3 h.

According to Table 11 samples showed a bacteriostatic effect depending on bacteria and time. It is noteworthy that the initial bacterial load of *E. faecalis* was approximately five times higher than that of *E. coli*, which may account for the observed differences in susceptibility to mollusk shell calcinated powders.

Notably, the samples obtained from *Rapana venosa* (Ca/CO/RP) and *Pecten maximus* (Ca/CO/SJ), which displayed the most intense CaO peaks and minimal residual carbonate, showed superior antibacterial efficiency—especially against *Enterococcus faecalis*, a Gram-positive species known for its high resistance to environmental stressors. The presence of portlandite, likely formed through post-calcination hydration of CaO, supports an antibacterial mechanism based on environmental alkalization. The resulting high pH (>12) promotes membrane destabilization and microbial inactivation. Furthermore, scanning electron microscopy (SEM) revealed porous, lamellar microstructures in these same samples, which contribute to increased surface area and facilitate ion release (Ca^2+^ and OH^−^), enhancing the bactericidal effect.

The differential susceptibility can be attributed to both the intrinsic structural characteristics of bacterial cell walls and the mechanism of action of calcium-oxide-based materials.

Gram-negative bacteria like *E. coli* possess a thin peptidoglycan layer (~2–7 nm) surrounded by an outer membrane rich in lipopolysaccharides (LPS). While this outer layer confers some protection against hydrophilic molecules and antibiotics, it is also susceptible to alkaline disruption and ionic imbalance induced by highly basic environments. The interaction with CaO or Ca(OH)_2_ particles elevates the local pH and generates hydroxide ions (OH^−^) that can destabilize the outer membrane, causing membrane depolarization, increased permeability, and eventual cell lysis [37,38,39].

In contrast, *E. faecalis*, a Gram-positive species, has a thick, multilayered peptidoglycan wall (~20–80 nm), which provides enhanced resistance to environmental stressors. The robust cell envelope is less susceptible to rapid pH-induced disruption and can maintain cellular integrity for longer under alkaline conditions. Additionally, *E. faecalis* is known for its high intrinsic stress tolerance, including resistance to desiccation, high salt, and pH fluctuations, which may delay its inactivation [32,34,37].

Moreover, the initial bacterial concentration used in the assay (approximately 500 CFU for *E. faecalis* vs. 100 CFU for *E. coli*) may have contributed to the observed differences. A higher initial load could require more time for the reactive species (e.g., hydroxyl ions or Ca^2+^) to achieve complete contact and disruption of all viable cells.

Thus, the enhanced sensitivity of *E. coli* to calcinated shell powders can be explained by its weaker physical barrier and greater susceptibility to alkaline and ionic stress, while the delayed inhibition of *E. faecalis* reflects its thicker cell wall and higher resistance to external chemical perturbation.

It is important to note that the interpretation of the antibacterial mechanism proposed in this study—based on the high alkalinity—is drawn from the analogous literature and not directly validated through specific assays. No ROS detection tests (e.g., DCFH-DA fluorescence), pH kinetics curves, or tests for microscopy evidence of bacterial membrane disruption were performed.

Together, these findings indicate that the antibacterial efficacy of biogenic-calcium-based materials is governed by their crystalline phase distribution, microstructural features, and reactivity. Therefore, the judicious selection of mollusk shell precursors and precise control over calcination conditions enable the tailoring of antimicrobial materials suitable for biomedical and environmental applications.

## 4. Discussion

Biogenic calcium carbonate derived from marine mollusk shells offers significant advantages over geological sources in terms of purity, sustainability, and potential for biomedical applications. Its high reactivity and fine structure make it a competitive alternative in specialized fields such as pharmaceuticals, cosmetics, and eco-materials. Although geological CaCO_3_ remains dominant in bulk industries due to its abundance, the valorization of marine shell waste presents a viable and environmentally friendly supplement or substitute for traditional sources.

The presented research investigates the characteristics of CaCO_3_ and CaO powders obtained by calcination of marine shells (*Chamelea gallina*, *Mya arenaria*, *Rapana venosa*, *Mytilus edulis*, and *Pecten maximus*) at 900 °C for 2 h. The results obtained were correlated between gravimetric yields, crystal composition, particle size, morphology, and colloidal stability.

Gravimetric yields ranged from 54.3% (raw material, *Chamelea gallina*) to 64.9% (raw material, *Pecten maximus*), indicating good efficiency of the conversion process. XRD analysis confirmed the predominant presence of CaO, but also traces of residual calcite and portlandite, the result of the reaction with moisture. ATR-FTIR supported these observations, showing characteristic bands for Ca–O and the elimination of organic groups.

The average particle size was between 15 μm (*Rapana venosa* raw material) and 37 μm (*Mytilus edulis* raw material), revealing particle size variations that directly influence the final applications. SEM showed sheet-like, folded, or fractured structures, reflecting both the biological nature of the source material and thermal transformations. These morphologies are essential for catalytic, filtration, or sorption applications.

The presence of CaO (lime) as the dominant phase across all samples indicates successful thermal decomposition of CaCO_3_, which enhances chemical reactivity due to the high surface energy and basic character of CaO. However, the detection of residual calcite in several samples (e.g., Ca/CO/CG, Ca/CO/MA, and Ca/CO/RP) suggests incomplete calcination, potentially reducing the reactivity and homogeneity of the final product. This incomplete transformation may be attributed to particle size variations, localized heat transfer inefficiencies, or intrinsic differences in the shell matrix. Additionally, the portlandite (Ca(OH)_2_) phase identified in all samples is a result of post-calcination hydration of CaO, and its presence can modify the reactivity by introducing secondary hydroxyl-based reactions in aqueous environments.

The morphological analysis further complements the understanding of functional behavior. Samples with high porosity, thin lamellar structures, and wrinkled or fractured surfaces (e.g., Ca/CO/RP and Ca/CO/CG) present increased surface area and accessibility to external reactants, favoring enhanced reactivity, particularly in catalytic or ion-exchange processes. In contrast, samples like Ca/CO/MY and Ca/CO/SJ, which exhibit more compact structures with smoother surfaces, are expected to be less reactive but mechanically more stable, making them suitable for structural or support materials in composites.

Furthermore, dimensional analysis highlights that particle size plays a dual role, where finer particles (e.g., Ca/CO/RP) offer a greater specific surface area for chemical interaction, enhancing reactivity, whereas coarser and more uniformly distributed particles (e.g., Ca/CO/MA and Ca/CO/SJ) tend to favor physical stability, affecting properties such as packing density, permeability, and mechanical cohesion.

Taken together, the combination of crystalline phase composition, microstructure, and particle size distribution directly governs the material’s performance. Highly porous and reactive powders (e.g., Ca/CO/RP) are advantageous in environmental remediation, catalysis, or sorbents, whereas denser, more structurally robust powders (e.g., Ca/CO/SJ) are better suited for construction materials, fillers, or ceramic matrices, where stability and strength prevail over chemical reactivity.

The thermal decomposition profiles indicate species-dependent behavior in terms of reaction kinetics and conversion efficiency. While all samples achieve significant decarbonation upon heating to 1000 °C, the extent and rate of transformation vary, with Ca/CO/MA and Ca/CO/RP exhibiting more gradual but complete two-step decomposition, and Ca/CO/CG showing a sharp initial degradation followed by limited secondary transformation. These findings highlight the influence of biogenic origin on thermal reactivity and final oxide yield.

The zeta potential analysis showed low values (<±15 mV), indicating the tendency of aggregation and sedimentation, which needs to be corrected for colloidal applications. The most promising results were observed for powdery samples from *Rapana venosa* and *Pecten maximus*, which combined small particle sizes, high yields, and increased reactivity.

Mollusk shell calcinated powders demonstrate significant antibacterial effects against *Escherichia coli*, even at short contact durations. For *Enterococcus faecalis*, complete bacterial inhibition was achieved only under prolonged exposure. These findings highlight the potential of obtained powders as antibacterial agents, especially in applications where sufficient contact time can be ensured for effective action against Gram-positive bacteria.

The antibacterial performance observed is likely driven by a combination of surface alkalinity, ionic exchange (Ca^2+^ release), and potential surface interactions at the micro- and nanostructural level, as evidenced by SEM images showing porous, wrinkled morphologies with large surface areas. These features are particularly favorable for enhancing contact between reactive materials and bacterial membranes, accelerating destabilization processes. Therefore, the mechanistic insights provided here should be viewed as hypothetical and require future confirmation through targeted biochemical and microbiological investigations. Future studies will include the BET method to more precisely correlate surface area with antibacterial efficacy.

Taken together, the results support the use of CaO-rich biogenic powders as effective antimicrobial agents, with performance being dependent on both material characteristics and microbial cell wall structure. The findings also underline the importance of exposure time and bacterial load in evaluating antibacterial efficacy, especially when comparing Gram-positive and Gram-negative species.

The complete conclusion is that biogenic synthesis from mollusk shells offers a sustainable and environmentally friendly alternative for obtaining CaO and CaCO_3_, with varied antibacterial impacts. Choosing the source material species and optimizing the calcination parameters are essential to adapt the properties of the powder to the requirements of the applications.

## Figures and Tables

**Table 2:** Comparative analysis of biogenic and geological CaCO_3_ synthesis methods.

Parameter	Biogenic CaCO_3_ (Marine Mollusk Shells)	Geologic CaCO_3_ (Rock)	References
Origin	Biological—formed by marine organisms such as mussels, scallops, clams, and snails	Geological—formed through sedimentation and diagenesis of marine deposits	[1,2,3,4,5,6,7,8,9,10,11,12,13]
Formation Process	Biomineralization: organisms secrete CaCO_3_ to build shells	Long-term accumulation of carbonate sediments, compression, and lithification	
Processing	Involves cleaning and thermal treatment (700–1000 °C)	Mining, crushing, and calcination (850–1100 °C)	
Purity	Can vary by species; may require removal of organic matter	Can contain silicates or other mineral impurities	
Particle Morphology	Typically lamellar, wrinkled, or hierarchical microstructures; varies by species and treatment	Irregular morphology; dependent on geological origin and grinding	
Ecological Impact	Reuses biological waste, supports circular economy, and low CO_2_ emissions during sourcing and calcination	Mining leads to habitat degradation, resource depletion, and high CO_2_ emissions	

**Table 3:** Comparative analysis of CaO synthesis methods.

Synthesis Method	Advantages of the Method	Limitations of the Method	References
Limestone calcination (conventional method)	Mature technology, wide industrial applicability, high yield	High energy consumption, high CO_2_ emissions, dependence on fossil natural resources	[28]
Sol–gel synthesis	Precise control over particle morphology and size, high purity	High costs of reagents, process sensitive to synthesis parameters	[14,15,18]
Coprecipitation	Relatively simple process, good yields, possibility of doping	Requires rigorous control of pH and reaction conditions	[13]
Decomposition of organo-metallic precursors	Enables composition-controlled nanoparticle synthesis	Precursors can be toxic or unstable, require strict conditions	[16]
Biogenic synthesis from mollusk shells	Recovery of marine litter, a product with a large specific area	Requires rigorous cleaning, composition may vary by species	[17,19]
Biogenic synthesis from animal bones	Reuse of animal waste, ecological synthesis, CaO with acceptable purity	Additional purification process, unpleasant odor emissions during calcination	[26]

**Table 4:** Sample coding and calcination parameters.

Marine Mollusk Shells	Initial Quantity (g)	Calcination Temperature (°C)	Calcination Time (h)	Final Quantity (g)	Calcination Yield (%)	Theoretic CaO Quantity (g)	Sample Codification
*Chamelea gallina*	20	900	2	10.857	54.285	6.088	Ca/CO/CG
*Mya arenaria*				10.936	54.679	6.124	Ca/CO/MA
*Rapana venosa*				11.232	56.16	6.298	Ca/CO/RP
*Mytilus edulis*				10.881	54.405	6.102	Ca/CO/MY
*Pecten maximus*				12.987	64.935	7.283	Ca/CO/SJ

**Table 5:** XRD data on standards and samples.

Sample Code	Phase	2Ɵ						
CaO	lime	32.2	37.4	53.8	67.2	79.3	-	
CaCO_3_	calcite	29.4	39.4	43.1	47.5	48.5	-	
	vaterite	24.9	27.0	32.7	43.8	49.1	55.7	
	aragonite	26.2	27.2	33.2	37	45.9	50.2	
Ca(OH)_2_	portlandite	18.0	34.0	47.1	50.8	54.3		
Ca/CO/CG	lime	32.35	37.44	54.00	64.37	67.33		
	calcite	28.74	47.3	60.32				
	portlandite	18.11	34.32	47.36	51.02	62.87	64.49	
Ca/CO/MA	lime	32.35	37.51	54.05	64.28	67.47		
	calcite	29.13	45.71	48.48	60.33			
	portlandite	18.14	28.72	34.32	47.32	50.92	62.8	64.48
Ca/CO/RP	lime	32.29	37.55	53.89	64.26	67.42		
	calcite	29.13	42.48	48.41	60.27			
	portlandite	18.15	28.86	34.28	47.30	50.92	62.76	64.43
Ca/CO/MY	lime	32.29	37.44	54.00	64.26			
	calcite	29.18	42.48	48.47	60.38			
	portlandite	18.11	28.9	34.28	47.36	51.07	62.76	64.43
Ca/CO/SJ	lime	32.29	37.44	53.89	64.26	67.43		
	calcite	28.96						
	portlandite	18.11	28.74	34.27	47.26	51.01	62.76	64.54

**Table 6:** Vibrational correlation of phases.

Phase	Wavenumber (cm^−1^)	Vibrational
CaCO_3_ (calcite)	~1400, ~870	CO_3_^2−^ (stretching, bending)
CaO (lime)	~500–350	Ca–O (metal-oxygen)
Ca(OH)_2_ (portlandite)	~3640	Stretching O–H

**Table 7:** Mean size of calcinated shells.

Sample	Marine Mollusk Shells	Mean Size (µm)
Ca/CO/CG	*Chamelea gallina*	20.1277
Ca/CO/MA	*Mya arenaria*	31.8910
Ca/CO/RP	*Rapana venosa*	15.0994
Ca/CO/MY	*Mytilus edulis*	36.9941
Ca/CO/SJ	*Pecten Maximus*	24.3651

**Table 8:** Thermal analysis parameters of Ca/CO samples derived from TGA thermograms.

Sample	1st Decomposition			2nd Decomposition			m_residuu_ (%)
	t_onset,1_ (°C)	t_max,1_ (°C)	Δm_1_ (%)	t_onset,2_ (°C)	t_max,2_ (°C)	Δm_2_ (%)	
Ca/CO/CG	387.96	418.43	23.18	576.50	621.48	6.44	69.49
Ca/CO/MA	387.92	430.57	16.20	622.33	696.45	17.13	66.28
Ca/CO/RP	395.07	437.35	15.85	620.64	695.70	17.02	66.82
Ca/CO/SJ	387.98	420.20	17.66	616.56	689.83	14.38	67.52
Ca/CO/MY	388.90	429.38	19.13	615.85	686.42	11.42	69.19

t_onset_—the onset temperature of the decomposition reaction; t_max_—the temperature at the minimum point of the 1st derivative of the TGA curve associated to the temperature of the maximum decomposition reaction rate; Δm—the mass loss in the corresponding decomposition process; m_residuu_—residue at 1000 °C.

**Table 9:** Zeta potential values of samples correlated with mean size.

Sample	Mean Size (µm)	Measurement	Temperature (°C)	pH	Viscosity (mPa/s)	Conductivity (mS/cm)	Electrophoretic Mobility (cm^2^/VS)	Zeta Potential (Mean) (mV)
Ca/CO/CG	20.1277	1	24.9	12.71	0.897	5.821	0.000015	2.0
		2	24.8		0.898		0.000028	3.6
		3	25.0		0.985		0.000014	1.8
Ca/CO/MA	31.8910	1	25.0	12.68	0.895	6.546	0.000006	0.8
		2	24.9		0.897		−0.000001	−0.1
		3	24.9		0.897		−0.000002	−0.2
Ca/CO/RP	15.0994	1	24.9	12.69	0.897	5.824	0.00028	3.6
		2	24.8		0.899		0.000102	13.3
		3	25.0		0.895		0.000053	6.9
Ca/CO/MY	36.9941	1	25.0	12.68	0.896	6.673	0.000016	2.0
		2	24.9		0.897		0.000015	1.9
		3	24.8		0.898		0.000017	2.2
Ca/CO/SJ	24.3651	1	24.8	12.70	0.898	5.605	0.00007	0.9
		2	25.0		0.896		0.000033	4.3
		3	25		0.894		0.000113	14.5

**Table 10:** Antibacterial activity of calcium-based materials.

Compound	Activity	Reference
CaO (from eggshells) + AgNPs	Strong antibacterial and photocatalytic activity; synergistic effect CaO–AgNPs against Gram-positive and Gram-negative bacteria	[30]
CaCO_3_ NP (from eggshells)	Antibacterial and antibiofilm activity against S. aureus and *E. coli*; mechanism dependent on size, pH, and Ca^2+^ ions	[31]
CaCO_3_ și α-TCP cu HA doped with Ag	Increases local pH, indirect antibacterial effect; main activity attributed to Ag^+^ ions	[32]
Ca(OH)_2_	Destroys biofilm of *E. faecalis*; affects morphology and dissolves the extracellular matrix	[33]
Ca(OH)_2_ + superoxidized solution	Synergistic activity against *E. faecalis*; increased antibacterial efficacy	[35]
Ca(OH)_2_ as liner dental	Inhibits bacterial growth under dental restorations through alkalization effect	[37]

**Table 11:** Reduction in the number of viable *E. coli* on the cultural environment CCA; and viable *E. faecalis* on the cultural environment SB.

Sample	*E. coli* Contact Time < 30 min. CFU/1 mL	*E. coli* Contact Time 3.3 h. CFU/1 mL	*E. faecalis* Contact Time < 30 min. CFU/1 mL	*E. faecalis* Contact Time 3.3 h. CFU/1 mL
Positive control (M^+^)	127 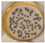	87 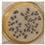	~500 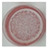	~500 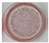
Negative control (M^−^)	0 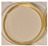	0 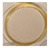	0 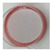	0 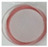
Ca/CO/CG	0 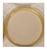	0 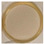	0 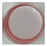	0 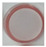
Ca/CO/MA	0 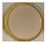	0 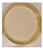	0 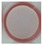	0 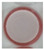
Ca/CO/RP	0 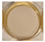	0 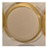	1 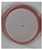	0 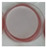
Ca/CO/MY	0 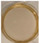	0 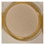	0 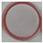	0 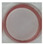
Ca/CO/SJ	0 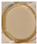	0 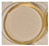	1 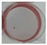	0 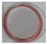

## Data Availability

The original contributions presented in this study are included in the article. Further inquiries can be directed at the corresponding author.
